# Surgical Management of Foveal Telangiectatic Capillaries in Diabetic Macular Edema

**DOI:** 10.7759/cureus.91026

**Published:** 2025-08-26

**Authors:** Hiroshi Tanaka, Kohsaku Numa, Hokoru Yoshioka, Takanori Aoki, Chie Sotozono

**Affiliations:** 1 Department of Ophthalmology, Kyoto Prefectural University of Medicine, Kyoto, JPN

**Keywords:** diabetic macular edema, fovea, surgical management, telangiectatic capillaries, telcaps, vitrectomy

## Abstract

We report a case of diabetic macular edema (DME) resistant to anti-VEGF therapy that was successfully treated with surgical intervention targeting foveal telangiectatic capillaries (TelCaps). A 61-year-old woman with a history of proliferative diabetic retinopathy and previous panretinal photocoagulation in both eyes developed refractory DME in the right eye, despite multiple intravitreal anti-VEGF injections. Indocyanine green angiography (ICGA) revealed two TelCaps located in the foveal and temporal regions. A 27-gauge pars plana vitrectomy was performed, including internal limiting membrane peeling around the TelCaps. The TelCaps were carefully grasped, elevated into the vitreous cavity, and treated by focal laser photocoagulation applied to their lateral surfaces using a curved laser probe, in order to minimize retinal damage. At 3 months postoperatively, the macular edema had resolved, best-corrected visual acuity improved from 20/100 to 20/33, and ICGA confirmed the absence of residual TelCaps. This case underscores the potential of surgical management as an alternative treatment for anti-VEGF-resistant TelCaps in patients with DME, particularly when the lesions are located in the fovea and are not amenable to conventional laser therapy.

## Introduction

Diabetic maculopathy, also known as diabetic macular edema (DME), is a major cause of vision loss in patients with diabetes [1]. It results from the breakdown of the blood-retinal barrier and accumulation of fluid in the macula, leading to retinal thickening and visual impairment. With the global increase in the prevalence of diabetes, the number of patients affected by vision-threatening DME is also expected to rise [2,3].

The first-line treatment for DME is intravitreal injection of anti-vascular endothelial growth factor (anti-VEGF) agents. However, some patients show resistance to therapy. Previous reports have suggested that factors such as high intraretinal reflectivity and a greater number of microaneurysms (MAs) and larger size of MAs are associated with poor treatment response [4,5]. Among these, large MAs with a diameter exceeding 150 µm - referred to as telangiectatic capillaries (TelCaps) [6] - have been identified as contributors to treatment resistance. TelCaps are often accompanied by hard exudates and retinal hemorrhages, leading to deterioration of vision. While laser photocoagulation is typically effective when TelCaps are located in non-central areas of the posterior pole [7], those forming within the macula may be more challenging to manage.

In a previous study, we reported for the first time the successful surgical removal of a TelCap located near the fovea secondary to branch retinal vein occlusion (BRVO) [8]. In this present study, we describe a case in which TelCaps located in the fovea and temporal region, secondary to DME, were surgically treated with favorable anatomical and functional outcomes.

## Case presentation

**Figure 1:**
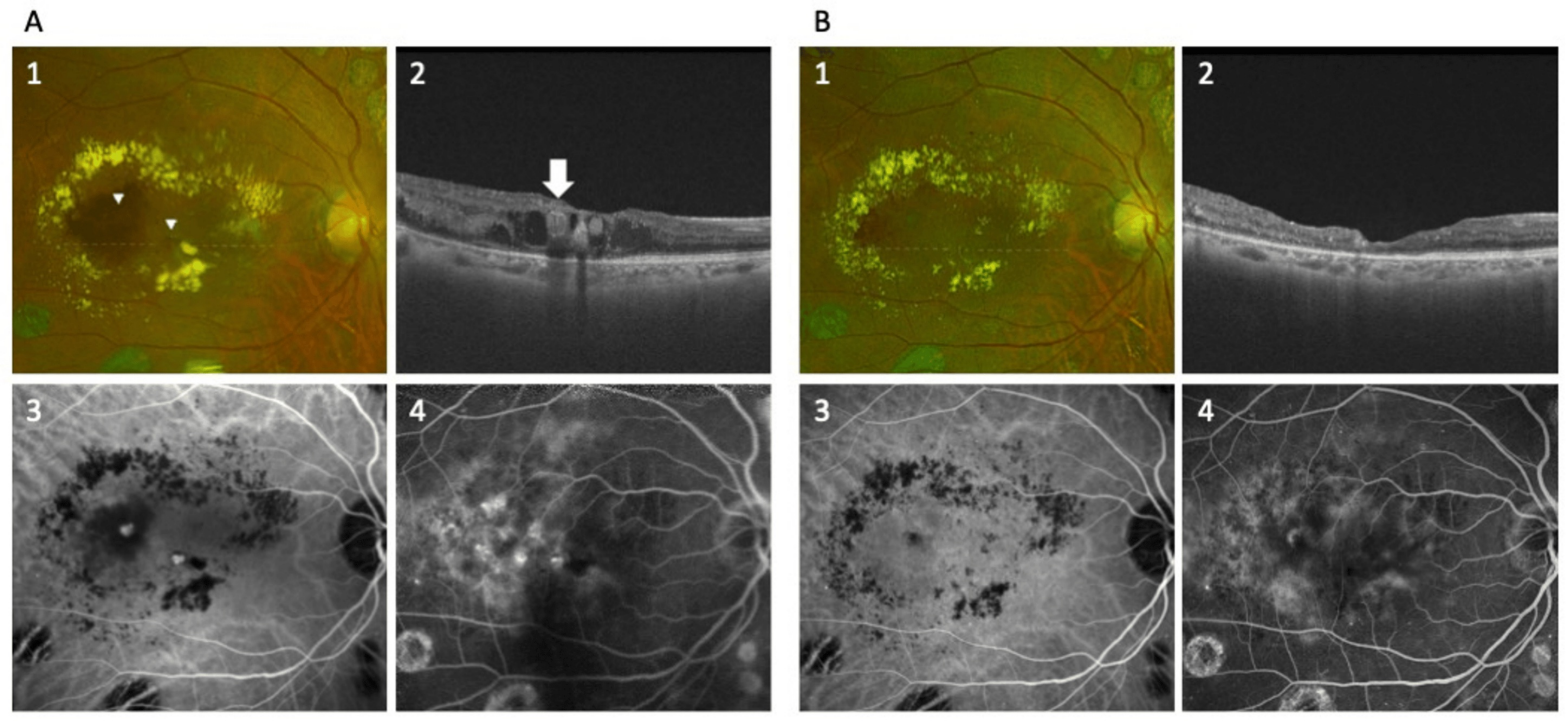
Pre- and postoperative multimodal imaging of TelCaps A) Images of the preoperative fundus findings. 1) Color fundus photography shows treatment-resistant telangiectatic capillaries (TelCaps) in the parafoveal and temporal regions, accompanied by retinal hemorrhages and surrounding hard exudates (HEs), with the TelCaps indicated by white arrowheads and the location of the OCT scan corresponding to A-2 shown as a white dotted line. 2) A horizontal cross-sectional optical coherence tomography (OCT) image reveals a ﻿high-intensity TelCaps in the fovea (arrow). 3, 4) ﻿Fundus angiography revealed TelCaps in the macula and temporal areas on late-phase indocyanine green angiography (ICGA) examination and fluorescent leakage around the TelCaps on late fluorescein angiography (FA) examination. B) Images of the fundus findings at three months postoperative. 1) Color fundus photography shows whitening of the TelCaps along with a reduction in retinal hemorrhage and HEs, with the location of the OCT scan corresponding to B-2 indicated by a white dotted line. 2) ﻿The closure of the TelCaps and resolution of macular edema were ﻿confirmed by horizontal OCT scan. 3, 4) ﻿Fundus angiography also confirmed closure of the TelCaps in the parafoveal and temporal regions on late-phase ICGA examination, and FA examination showed decreased late fluorescence leakage around the TelCaps.

A 61-year-old woman with a history of proliferative diabetic retinopathy had undergone panretinal photocoagulation in both eyes and was pseudophakic bilaterally. She developed DME in the right eye and received a total of nine intravitreal aflibercept (2 mg) injections, seven times over a 10-month period between November 2023 and September 2024, with the final injection administered in September 2024, three months prior to surgery. Despite repeated treatments, the outcome was poor, and she was referred to the Department of Ophthalmology at Kyoto Prefectural University of Medicine, Kyoto, Japan. At presentation, best-corrected visual acuity (BCVA) in the right eye was 20/100. Fundus examination revealed macular edema, circinate exudates, and retinal hemorrhage. OCT examination demonstrated the TelCap at the fovea, in which the lumen was filled with reflective material and surrounded by a hyperreflective outer wall, along with associated edema and retinal hemorrhage. Fluorescein angiography (FA) showed diffuse leakage, and indocyanine green angiography (ICGA) revealed two large microaneurysms located in the fovea and temporal region, consistent with TelCaps (Fig. 1A). After thorough explanation of the benefits and risks of our novel surgical approach for TelCaps, written informed consent to undergo surgery was obtained from the patient in December 2024.

**Figure 2:**
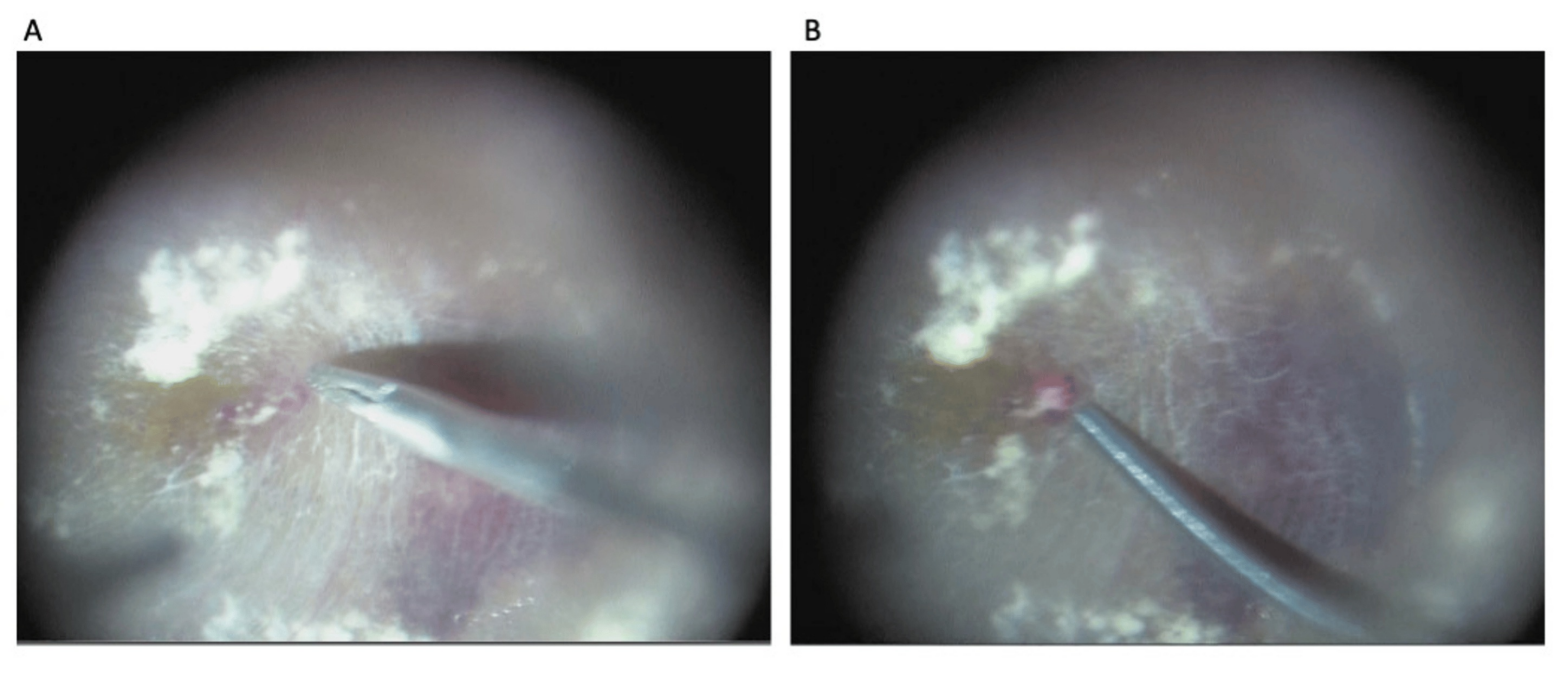
Images of the intraoperative findings A) After incising the inner retina, the TelCaps were grasped with 27-gauge microforceps and dissected from the surrounding tissue. B) Intraoperative endolaser focal photocoagulation was performed on the lateral surface of the TelCaps in the vitreous cavity using a curved laser.

Briefly, a standard 27-gauge (G) pars plana vitrectomy using the CONSTELLATION® Vision System (Alcon Laboratories, Inc., Fort Worth, TX, USA) with a wide-angle fundus-viewing system (RESIGHT®; Carl Zeiss Meditec AG, Jena, Germany) was performed under sub-Tenon’s anesthesia with approximately 5 mL of 2% lidocaine. First, internal limiting membrane peeling was performed in the area surrounding the TelCaps using 27-G microforceps (DEX™ Super Grip Forceps; Katalyst™ Surgical, Chesterfield, MO, USA) with magnifying contact lens (Hoya Corporation, Tokyo, Japan), after visualization of the ILM with triamcinolone acetonide (MaQaid®, Wakamoto Pharmaceutical Co., Tokyo, Japan). Next, the superficial retinal layers over the two TelCaps were gently incised, and the exposed TelCaps were directly grasped with the 27-G microforceps and lifted into the vitreous cavity while separated from the surrounding tissue. Intraoperative focal laser photocoagulation (see Video 1) was performed to produce a grayish-white burn of the TelCaps (i.e., a total of 26 shots; duration: 0.10 seconds per shot; power: 80 mW) with a 27+ Flex-Tip Laser Probe (Alcon Laboratories), including six shots applied to the TelCaps located at the fovea (Fig. 2). Laser treatment was applied from the side of the lifted TelCaps to ensure thermal occlusion while avoiding damage to adjacent tissues. Finally, air exchange was performed, and the surgery was completed after confirming the closure of the wound without sutures.

**Video 1:** Intraoperative maneuvers for TelCaps A standard 27-gauge pars plana vitrectomy is performed with wide-angle viewing system. Internal limiting membrane (ILM) peeling is first conducted around the TelCaps using 27-G microforceps under high magnification provided by a contact lens. The superficial retinal layers are then incised to expose the TelCaps, which are grasped and lifted into the vitreous cavity. Focal endolaser photocoagulation is applied to the lateral surface of the TelCaps, inducing thermal closure while preserving surrounding tissue. The procedure is completed with air exchange.

From the first day postoperative, no intraocular pressure increase, intraocular inflammation, or vitreous hemorrhage was observed. At three months postoperative, fundus examination showed a reduction in hard exudates and retinal hemorrhages, and OCT revealed occlusion of the TelCap and resolution of macular edema. BCVA improved to 20/33. Follow-up FA showed reduced late-phase leakage, and ICGA revealed no detectable TelCaps (Fig. 1B), indicating successful occlusion and improved retinal function resulting from the surgical intervention.

## Discussion

In this report, we presented a case of DME resistant to anti-VEGF therapy in which multiple TelCaps located in the foveal and temporal regions were successfully treated using a novel surgical approach, thus leading to resolution of the macular edema and improvement of BCVA. Previously, we reported a similar approach in a case of a BRVO-associated parafoveal TelCap that was resistant to anti-VEGF therapy [8], which also resulted in favorable anatomical and functional outcomes.

The efficacy of anti-VEGF therapy for TelCaps has been reported to vary depending on lesion size. While smaller TelCaps tend to regress with anti-VEGF treatment, larger ones often persist despite repeated injections. In light of this background, a treatment strategy has been proposed in which residual TelCaps are selectively targeted with laser photocoagulation. Indeed, a multicenter prospective study is currently ongoing to compare the efficacy of anti-VEGF monotherapy versus combination therapy with photocoagulation in patients with TelCap-associated macular edema. However, that study excludes lesions located within 500 µm of the foveal center [9].

Treatment of TelCaps located near the fovea, as in the present case, remains a therapeutic challenge and represents an "unmet medical need." Our surgical approach may provide a promising alternative option for such treatment-resistant lesions.

A key advantage of this technique is the ability to coagulate the TelCaps from the side after lifting them onto the retinal surface using a curved laser probe. This allows for targeted photocoagulation while minimizing thermal damage to the outer retinal layers, even in anatomically sensitive regions. However, the procedure involves mechanical manipulation, i.e., grasping and lifting the TelCap, which carries a risk of iatrogenic trauma and submacular hemorrhage. Therefore, careful preoperative and postoperative assessment is essential. In cases with a high risk of submacular hemorrhage, the adjunctive use of intravitreal tPA at the conclusion of surgery with gas tamponade may be considered to facilitate hemorrhage resorption.

## Conclusions

This case demonstrates that surgical treatment with endolaser photocoagulation for treatment-resistant TelCaps located within the fovea and temporal macula can achieve successful anatomic closure, resolution of diabetic macular edema, and significant visual improvement. Our novel approach enabled safe and targeted coagulation of TelCaps in anatomically critical regions, where conventional laser photocoagulation is not feasible. Although further studies with larger case series and longer follow-up are required, this technique may represent a promising therapeutic option for patients with vision-threatening DME harboring treatment-resistant TelCaps.

## References

[1] Shah J, Nguyen V, Hunt A (2022). Characterization of poor visual outcomes of diabetic macular edema: the fight retinal blindness! Project. Ophthalmol Retina.

[2] (2021). Causes of blindness and vision impairment in 2020 and trends over 30 years, and prevalence of avoidable blindness in relation to VISION 2020: the Right to Sight: an analysis for the Global Burden of Disease Study. Lancet Glob Health.

[3] Teo ZL, Tham YC, Yu M (2021). Global prevalence of diabetic retinopathy and projection of burden through 2045: systematic review and meta-analysis. Ophthalmology.

[4] Lee J, Moon BG, Cho AR, Yoon YH (2016). Optical coherence tomography angiography of DME and its association with anti-VEGF treatment response. Ophthalmology.

[5] Yamada Y, Takamura Y, Morioka M, Oshima H, Gozawa M, Matsumura T, Inatani M (2024). Characteristics of microaneurysm size in residual edema after intravitreal injection of faricimab for diabetic macular edema. J Clin Med.

[6] Castro Farías D, Matsui Serrano R, Bianchi Gancharov J (2020). Indocyanine green angiography for identifying telangiectatic capillaries in diabetic macular oedema. Br J Ophthalmol.

[7] Paques M, Philippakis E, Bonnet C (2017). Indocyanine-green-guided targeted laser photocoagulation of capillary macroaneurysms in macular oedema: a pilot study. Br J Ophthalmol.

[8] Tanaka H, Kojima K, Miyatani T, Kusada N, Terao N, Nagata K, Sotozono C (2024). A new surgical approach for the treatment of a refractory foveal microaneurysm: a case report. Am J Ophthalmol Case Rep.

[9] Dupas B, Castro-Farias D, Girmens JF (2024). Photocoagulation or sham laser in addition to conventional anti-VEGF therapy in macular edema associated with TelCaps due to diabetic macular edema or retinal vein occlusion (TalaDME): a study protocol for a multicentric, French, two-group, non-commercial, active-control, observer-masked, non-inferiority, randomized controlled clinical trial. Trials.

